# Research to classrooms: a co-designed curriculum brings *All of Us* data to secondary schools

**DOI:** 10.1093/jamia/ocae167

**Published:** 2024-07-09

**Authors:** Louisa A Stark, Kristin E Fenker, Harini Krishnan, Molly Malone, Rebecca J Peterson, Regina Cowan, Jeremy Ensrud, Hector Gamboa, Crstina Gayed, Patricia Refino, Tia Tolk, Teresa Walters, Yong Crosby, Rubin Baskir

**Affiliations:** Genetic Science Learning Center, Department of Human Genetics, University of Utah, Salt Lake City, UT 84112, United States; Genetic Science Learning Center, Department of Human Genetics, University of Utah, Salt Lake City, UT 84112, United States; Genetic Science Learning Center, Department of Human Genetics, University of Utah, Salt Lake City, UT 84112, United States; Genetic Science Learning Center, Department of Human Genetics, University of Utah, Salt Lake City, UT 84112, United States; Genetic Science Learning Center, Department of Human Genetics, University of Utah, Salt Lake City, UT 84112, United States; Science Department, Mojave High School, North Las Vegas, NV 89031, United States; Science Department, Canby High School, Canby, OR 97013, United States; Science Department, West Middle School, Bay Shore, NY 11706, United States; Science Department, Technology High School, Paramus, NJ 07652, United States; Science Department, East Rockaway High School, East Rockaway, NY 11518, United States; Science Department, Lincoln High School, Sioux Falls, SD 57105, United States; Science Department, Loup City High School, Loup City, NE 68853, United States; Division of Engagement and Outreach, All of Us Research Program, National Institutes of Health, Bethesda, MD 20817, United States; Division of Engagement and Outreach, All of Us Research Program, National Institutes of Health, Bethesda, MD 20817, United States

**Keywords:** big data, curriculum, educational assessment, education, NIH *All of Us* Research Program

## Abstract

**Objectives:**

We describe new curriculum materials for engaging secondary school students in exploring the “big data” in the NIH *All of Us* Research Program’s Public Data Browser and the co-design processes used to collaboratively develop the materials. We also describe the methods used to develop and validate assessment items for studying the efficacy of the materials for student learning as well as preliminary findings from these studies.

**Materials and Methods:**

Secondary-level biology teachers from across the United States participated in a 2.5-day Co-design Summer Institute. After learning about the *All of Us* Research Program and its Data Browser, they collaboratively developed learning objectives and initial ideas for learning experiences related to exploring the Data Browser and big data. The Genetic Science Learning Center team at the University of Utah further developed the educators’ ideas. Additional teachers and their students participated in classroom pilot studies to validate a 22-item instrument that assesses students’ knowledge. Educators completed surveys about the materials and their experiences.

**Results:**

The “Exploring Big Data with the *All of Us* Data Browser” curriculum module includes 3 data exploration guides that engage students in using the Data Browser, 3 related multimedia pieces, and teacher support materials. Pilot testing showed substantial growth in students’ understanding of key big data concepts and research applications.

**Discussion and Conclusion:**

Our co-design process provides a model for educator engagement. The new curriculum module serves as a model for introducing secondary students to big data and precision medicine research by exploring diverse real-world datasets.

## Introduction

The next generation of tools for advancing health have arrived in the form of big data. A key example is the National Institutes of Health’s (NIH) *All of Us* Research Program (*All of Us*).^1^  *All of Us* is a landmark, longitudinal initiative that is partnering with at least 1 million individuals residing in the United States who consent to sharing health data, with the goal of advancing research and improving health.^2^ By providing access to a dataset that allows researchers to connect factors such as lifestyle, socioeconomics, environment, and genetics, the program aspires to pave the way for further advances in precision medicine—tailored prevention, treatment, and care strategies—for all of us.

*All of Us* is dedicated to fostering a participant community that mirrors the rich diversity found within the United States. Its strategy for cultivating a varied database has been purposeful and driven by engagement with participants and their communities, particularly in light of disparities across multiple communities in biomedical research.^3^ Using this strategy, *All of Us* has built a large and diverse cohort. Among the 776 000+ individuals (as of March 12, 2024) who have started the initial steps of the program’s protocol, a notable 80%+ self-identify as belonging to underrepresented groups within biomedical research, including diversity categories for race and ethnicity, gender identity, sex assigned at birth, sexual orientation, disability, and others.^4^ Of these, nearly half self-identify as belonging to racial and ethnic minority groups.

To ensure data privacy and security, the program uses a tiered structure of data access, requiring training to access more sensitive data.^5^ The Public tier, which does not have a training requirement, includes a Data Browser which allows users to use search terms to review de-identified, aggregate information about the *All of Us* cohort.^6^

The *All of Us* program is committed to workforce diversity and training with the aim of supporting researchers who span career stages, demographics, institutional settings, geographies, and disciplines.^6^ To address this goal, the program focuses on 5 key researcher audiences: (1) K-12 students and teachers; (2) undergraduate, graduate, and medical students; (3) post-doctoral students and early stage investigators; (4) established investigators; and (5) community and citizen scientists. The inclusion of both K-12 students and teachers is based on the understanding that engaging these early STEM students in developing and using the next generation of research tools will also require engaging with the educators of the next generation of researchers.^1^ The transparent nature of the program, the broad availability of its dataset, the self-contained nature of analysis within a centralized location, and the presence of a support infrastructure make the *All of Us* dataset a powerful resource for educators.^6^ Given the potential impact precision medicine may have on public health, an “early and often” education approach may be warranted.^7^

Developing lesson materials for secondary students that utilize the *All of Us* Data Browser provides an avenue for building their computational thinking skills. These curricula also can provide experience in working with large datasets to ask and answer research questions. Webb et al.^8^ note the emerging consensus about the importance of exposing students to key computational thinking concepts in K-12 education, arguing that early exposure is essential for building self-efficacy, particularly among girls, and thus promotes gender diversity in university computer science programs and the IT industry. However, teachers often lack understanding and awareness of concepts such as “Data Science,” “Data Analysis,” and “Data Mining,”^9^ which may limit their ability to develop lessons. By focusing on big data (eg, datasets, population-based data, aggregated samples, and databases), lesson materials utilizing the *All of Us* Data Browser can bridge this gap and equip teachers and students with the skills necessary to interpret and contextualize data in an increasingly digital world.

“Big data” plays an increasing role in our lives, from algorithms that determine what content appears in our browser searches or social media feed to routing for delivery vehicles. Introducing secondary students to big data in the context of health provides opportunities to educate them about the value of large datasets, engage them in research, and spark interest in biomedical research careers. High school also is a critical time to engage students since it is the last time most people in the United States study the subjects of biology or health.

Teachers are the gatekeepers to the instructional materials used in classrooms. Collaborating with them to co-design curriculum materials helps ensure that the lessons will fit within the scope and sequence of courses and are at an appropriate level for the targeted grade(s). Bringing together educators who teach in a range of settings with students from differing backgrounds can inform development of curricula that will be broadly used.^10^

## Objectives

Here, we describe the methods used to facilitate secondary teachers’ collaboration in co-designing curriculum materials focused on engaging students in exploring *All of Us* data and to further develop these materials. We also describe the methods that were employed to develop and validate the assessment items that will be used to study the efficacy of the curriculum materials for student learning. Finally, we report preliminary findings related to our research question: To what extent do the new curriculum materials improve students’ knowledge of “big data” and its application via the *All of Us* Research Program’s Data Browser?

## Methods

### Ethics approval and consent to participate

Prior to conducting research with teachers and students, the studies were determined to fall under human subjects exemption category 1 (conducted in an educational setting using normal educational practices) and category 2 (uses educational tests, surveys, interviews, or observations of public behavior) by the Institutional Review Board for the *All of Us* Research Program (RCB-2023-E007). Prior to classroom testing, we obtained research approval from each school’s principal and from the school districts that require this approval. We acquired written informed consent for study participation from all teachers who participated in the classroom testing and from students’ parents or guardians. Before classroom instruction began, teachers read an informational document about the study to students, who assented to participate by taking the assessment. Written informed assent was obtained from students who participated in think-aloud cognitive interviews.

### Curriculum co-design and classroom testing processes

**Figure 1.**
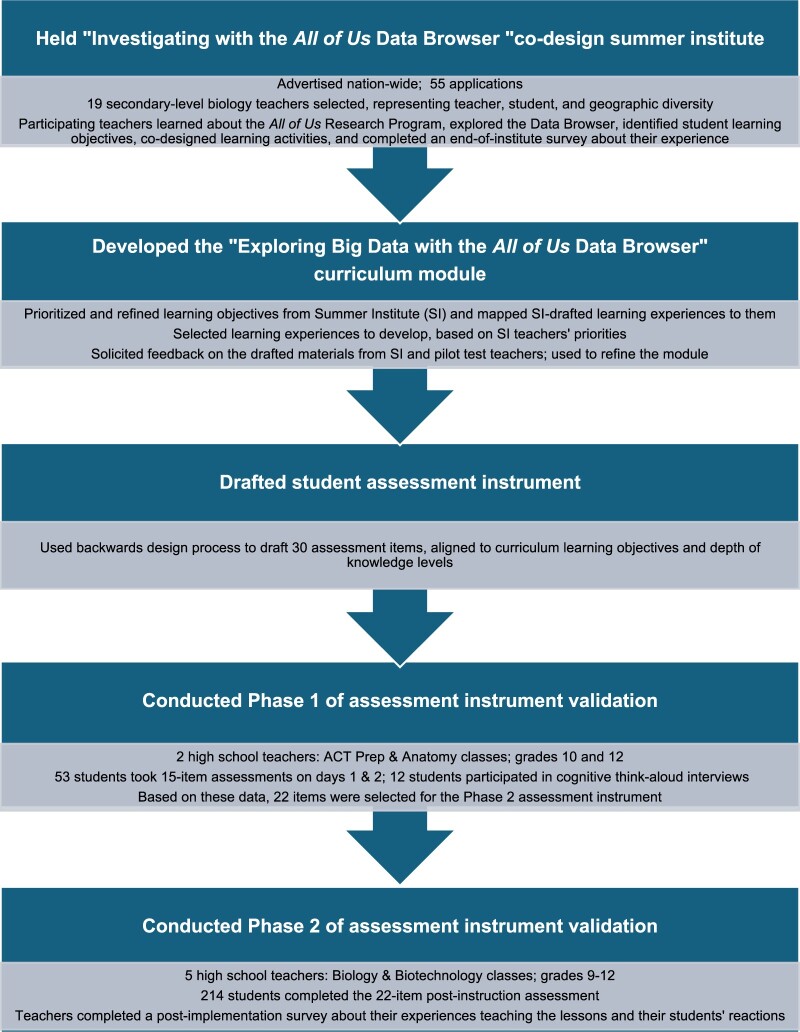
Processes for developing the “Exploring Big Data with the *All of Us* Data Browser” curriculum module and developing and validating the assessment instrument that will be used to study the efficacy of the module for student learning.

The processes used to co-design the “Exploring Big Data with the *All of Us* Data Browser” curriculum module are outlined in Figure 1. This figure also summarizes the processes used to develop and validate the assessment instrument that will be used to study the efficacy of the module for student learning. The processes are described in more detail in the following sections.

### Co-designing curriculum materials with secondary teachers

**Figure 2.**
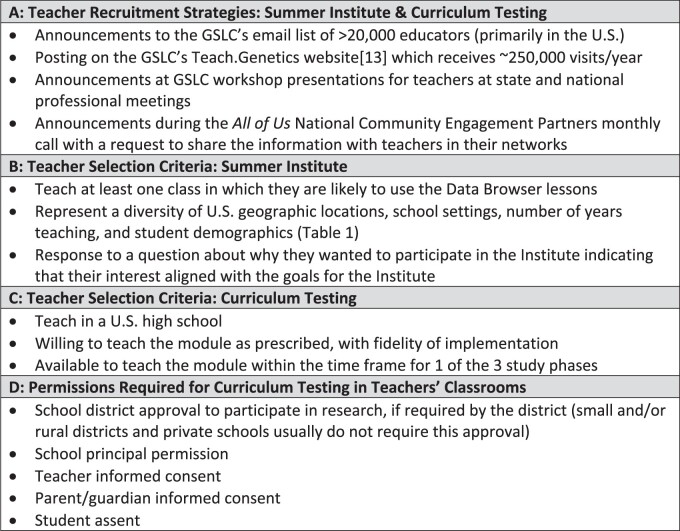
(A) Teacher recruitment strategies for participation in the Co-design Summer Institute and curriculum testing. (B) Teacher selection criteria for Summer Institute participation. (C) Teacher selection criteria for curriculum testing. (D) List of permissions required before selected teachers can participate in testing the curriculum module in their classroom(s).

The University of Utah’s Genetic Science Learning Center (GSLC) began the curriculum development process by holding a 2.5-day Co-design Summer Institute with secondary teachers from across the U.S. Recruitment strategies and the selection criteria for Institute participants are outlined in Figure 2. Since most of the curricula the GSLC develops and disseminates via its Learn.Genetics^11^ and Teach.Genetics^12^ websites focus on life science, the Center is primarily known by biology educators, who comprised the applicants.

A total of 55 educators from across the country applied to participate in the Summer Institute. Demographics for the 19 secondary-level biology teachers who were selected to participate and for the students in their schools are listed in Table 1. One of the teacher authors (J.E.) had participated in 2 prior summer institutes, enabling him to serve as a peer mentor in his working group during this institute.

**Table 1.** Demographics of teachers who participated in the Co-design Summer Institute and of students in the schools in which they teach.

Teacher demographics	Percentage and Number
**U.S. census region**	
South	16% (3)
Midwest	21% (4)
West	26% (5)
Northeast	37% (7)
**School setting**	
Urban	6% (1)
Rural	21% (4)
Suburban	47% (9)
**Number of years teaching**	
1-10 years	32% (6)
11-20 years	42% (8)
21-30 years	21% (4)
31-40 years	5% (1)

**Student demographics**	

**Race/ethnicity other than White, not Hispanic or Latino**	
0%-20%	21% (4)
21%-40%	26% (5)
41%-60%	21% (4)
61%-89%	5% (1)
81%-100%	26% (5)
**Eligible for free or reduced-price lunch**	
0%-20%	16% (3)
21%-40%	0
41%-60%	42% (8)
61%-89%	11% (2)
81%-100%	32% (6)

The Summer Institute utilized the GSLC’s established co-design processes.^10^ It began with an introduction to the *All of Us* Research Program and an orientation to the Data Browser by program representatives. Next, participants explored the Data Browser and self-selected into small working groups organized by data sources in the Data Browser or related areas of interest. Each working group generated learning objectives and initial ideas for learning experiences that utilize the Data Browser and connect with disciplinary core ideas and science practices in the Next Generation Science Standards.^13^ The initial ideas were subjected to a round of peer review, refined, and presented to the whole group at the end. *All of Us* representatives were available in-person and virtually to answer questions throughout. At the end of the Institute, participants completed a survey about their experience, responding to Likert scale and open-ended questions.

### Module development

GSLC team members with expertise in K-12 education, instructional design, science research, and writing reviewed the learning objectives and initial ideas for learning experiences generated by teachers during the institute. They first created a priority list of learning objectives based on generalizability among life science, health, and research classes and then worked with the GSLC’s research and evaluation team to refine these learning objectives for assessment and materials development. Next, the teams identified the ideas for learning experiences that closely mapped to the refined learning objectives, discussed the merits of each, and identified similarities or overlapping areas. They selected ideas to develop for the module based on the following criteria: flexibility for use in a variety of life science and health-related courses, modality (eg, video, web-based interactive, hands-on activity), feasibility for development, a lack of prerequisite knowledge or co-dependencies, and the appropriateness of student time-on-task to achieve the learning objective. These criteria were created in response to Institute participants’ request that the materials be usable in multiple subject areas, complement different points of entry into an instructional sequence, and span a reasonable amount of instructional time in light of existing required learning targets and constraints on instructional time to address them. Once the teams selected the learning activities to develop, the ideas were reviewed by the art, design, and multimedia team for feasibility. The materials were then developed through iterative rounds of writing and multimedia development.

### Teacher feedback on the curriculum materials

The GSLC solicited teacher feedback for refining the materials in 2 ways. They sent an online survey to the teachers who participated in the Co-design Summer Institute asking them to (a) rate the usefulness of each activity for their teaching and (b) provide details explaining their ranking. They also asked the classroom pilot test teachers to complete a post-instruction survey, adapted from previously validated GSLC teacher surveys.^14^^,^^15^ This survey gathered feedback about educators’ experiences teaching the lessons, any issues they or their students encountered, and their observations of students' reactions to the lessons. Quantitative data from the surveys were analyzed using descriptive statistics and qualitative data were analyzed using thematic analysis. Data from the surveys were used to inform revisions and improvements to the curriculum materials.

### Student assessment instrument development and validation

The GSLC’s research and evaluation team developed a student assessment that was designed to evaluate students' mastery of the learning objectives and depth of knowledge associated with the curriculum. Assessment item development followed a backwards design process,^16^ beginning with defining the learning objectives and their associated Depth of Knowledge levels^17^^,^^18^ for the Data Browser curriculum materials. Thirty assessment items were then drafted to align with these objectives and levels.

To validate the assessment instrument prior to its use in a national field study, the team employed a 2-phase process. The purpose of Phase 1 was to narrow the initial pool of assessment items from 30 down to 20-25 well-functioning items. Phase 2 of the assessment validation process aimed to assess the functionality and alignment of the selected items.

The GSLC recruited high school teachers to participate in the validation and field studies using the methods outlined in Figure 2. This figure also lists the selection criteria and the permissions that needed to be obtained for the selected teachers to test the module with their students. Teachers were assigned to participate in Phase 1 or 2 of the pilot study or the field study based on the dates they could teach the module. Descriptors for the participating classes, schools, and number of participating students are summarized in Table 2.

**Table 2.** Descriptors for students and schools participating in the Phase 1 and Phase 2 pilot tests of the *All of Us* Data Browser curriculum materials.

	Phase 1 pilot test		Phase 2 pilot test				
**Class name**	ACT Prep	Anatomy	Biology	Biology	Biology	Biotechnology	Biotechnology
**Class type**	General	Honors	General & Honors	General	General	General	Honors
**Grade level**	10th	12th	9th	10th	10th	11th & 12th	12th
**Number of students with scorable assessments**	20	33	19	42	42	94	19
**U.S. census region**	West	Midwest	Northeast	South	South	Midwest	West
**School setting**	Rural	Rural	Rural	Suburban	Suburban	Suburban	Suburban
**Students qualifying for free or reduced-price lunch**	81%-100%	21%-40%	61%-80%	0%-20%	0%-20%	0%-20%	0%-20%
**Race and ethnicity of students in the participating schools**							
American Indian or Alaska Native	40%	0%	0%	0%	0%	0%	0%
Black or African American	0%	0%	16%	4%	4%	16%	3%
Asian	0%	0%	4%	7% (reported together)	7% (reported together)	0%	28%^a^
Native Hawaiian or Other Pacific Islander	0%	0%	0%			0%	0%
White, Hispanic or Latino	8%	0%	8%	2%	2%	5%	12%
White, not Hispanic or Latino	50%	94%	72%	71%	71%	67%	39%
Others^b^	2%	6%	0%	0%	0%	5%	5%
Multiracial	0%	0%	0%	16%	16%	8%	13%

We counted an assessment as scorable if the student answered 50% or more of the questions.

a Includes Chinese, Korean, Japanese, and Vietnamese.

b The percentage of students representing each race or ethnicity included in this category was <1%; thus, they were aggregated as “Others.” May include American Indian or Alaska Native, Asian, Black or African American, Hispanic or Latino, Native Hawaiian or Other Pacific Islander, or 2 or more races.

Two teachers and their students participated in the Phase 1 pilot with students participating in 1 of 2 ways after using the materials: taking the assessment as an “online test” or participating in think-aloud cognitive interviews.^19^ For the “online test,” the assessment was divided into day 1 content (Form A) and day 2 content (Form B), each with 15 questions that were administered at the end of that day’s materials. Except for the students participating in the think-aloud cognitive interviews, all students took both test forms (A and B). This approach avoided complications due to the length of time and cognitive load that would have been associated with students taking all 30 assessment items at once. For the think-aloud cognitive interviews, each teacher was asked to invite 2 low-performing, 2 mid-performing, and 2 high-performing students to participate. These 6 students were then divided into 2 groups of 3 students with each triad consisting of 1 low-performing, 1 mid-performing, and 1 high-performing student. Each triad was assigned to 1 of the 2 test forms (either A or B). During a recorded Zoom interview, students in the triad were asked to “think-aloud” as they responded to the assessment items, providing insights into any ways in which the items might be confusing or misunderstood. The interviews were conducted by an experienced, independent consultant, as required by the IRB. After analyzing the data from these processes, 22 questions were selected for the single assessment instrument used in Phase 2.

Phase 2 of the assessment validation process aimed to assess the functionality and alignment of the selected items. Five teachers from across the United States (Table 2) implemented the *All of Us* Data Browser curriculum within their classes and administered the 22-item assessment to their students.

Stringent privacy measures were maintained throughout the validation process. Assessments did not impact students' grades, and all data collected were anonymized. Researchers did not interact directly with students and did not have access to any personally identifying information.

## Results

### Co-design Summer Institute

During the Institute, teachers self-selected into 7 working groups based on the “types” of data found in the Data Browser—electronic health records, surveys, genomics, and measurements and wearables—and by more specialized areas of interest—big data, triangulation in research, and health outcomes by sex assigned at birth. The groups outlined ideas for learning experiences in several modalities including short introductory videos, web-based interactive multimedia pieces, and paper-based activity guides to be used in conjunction with the Data Browser. In addition, the groups created larger “lesson plans” that interweaved their learning experience ideas with existing assets produced by the *All of Us* Research Program. Commonalities in ideas generated across the working groups included: short introductory videos that explore the concept of big data, web-based experiences that explain and explore the types of data the *All of Us* Research Program collects, and worksheets with prompting questions that guide students in exploring the Data Browser and using it to answer a research question.

On the end-of-Institute survey, slightly more than half of the participating teachers (56%) agreed that the 2.5-day Institute “was the right length,” while 33% somewhat agreed and 11% somewhat disagreed. Most participants (78%) were satisfied with the amount of time spent co-designing activity ideas in small groups. One of these participants noted that their group “needed more time but [not having more] also allowed us to scale down [ideas].” Another participant commented that there was “not enough time to fully flesh out the ideas.” Because participants self-selected the topic on which they wanted to focus their co-design, there was variation in group size (1-5 teachers/group). One recommendation was to have smaller groups (∼3 individuals) and to lengthen the Institute to allow more time for refining ideas. Nearly all (94%) respondents preferred the in-person Institute format noting that “online collaboration is harder.”

The survey asked respondents to assess the benefits of the Data Browser for classroom instruction. Most (72%) strongly agreed with the statement that “it is beneficial to develop classroom materials on the *All of Us* Data Browser” and 22% somewhat agreed. Conversely, 39% strongly agreed that “the *All of Us* Data Browser is a worthwhile teaching tool” and 56% somewhat agreed.

#### “Exploring big data with the All of Us Data Browser” curriculum module

While large, diverse datasets are valuable research tools,^20^^,^^21^ public perceptions often hold “big data” in a negative light.^22^ Additionally, critical thinking and data-intensive skills are requirements of many careers today, and there is a need for academic institutions to provide a range of pathways into these careers outside of 4-year or graduate degrees.^23^ Thus, we chose topics and activities that give students an accessible, early experience as data scientists and that showcase positive, real-world examples of data in action. The result is a flexible, high-school level curriculum module titled “Exploring Big Data with the *All of Us* Data Browser.”^24^ The module includes a mix of print and multimedia activities. Suggested learning sequences within the teacher support materials illustrate ways the materials can be useful in a variety of class settings, from the social sciences to biology and health.

**Figure 3.**
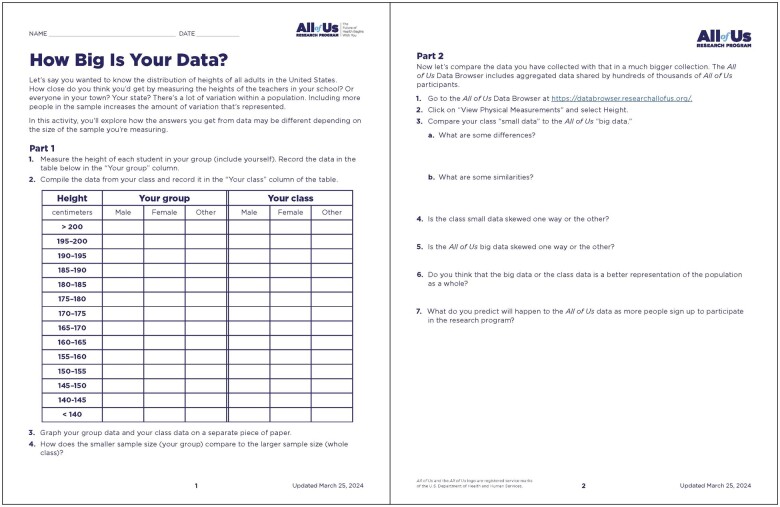
In the “How Big is Your Data?” activity, students collect, analyze, and interpret data, and compare small local datasets (small group and classroom data) to the larger amount of data available in the *All of Us* Data Browser.

Three print activities engage students in using the Data Browser. The “Data Browser Webquest” introduces students to the Data Browser and the types of available data. In “How Big is Your Data?” (Figure 3), students are introduced to considerations for gathering data while protecting participant privacy. They collect, analyze, and interpret data during an exercise in which they compare small local datasets (small group and classroom data) to the larger amount of data available in the Data Browser. In “Research with the Data Browser,” students make a prediction about whether several health conditions will be more prevalent in individuals whose sex assigned at birth was male, female, or other. They then record data from the Data Browser, calculate prevalence in each sex, and consider the strength of several potential questions for further research.

**Figure 4.**
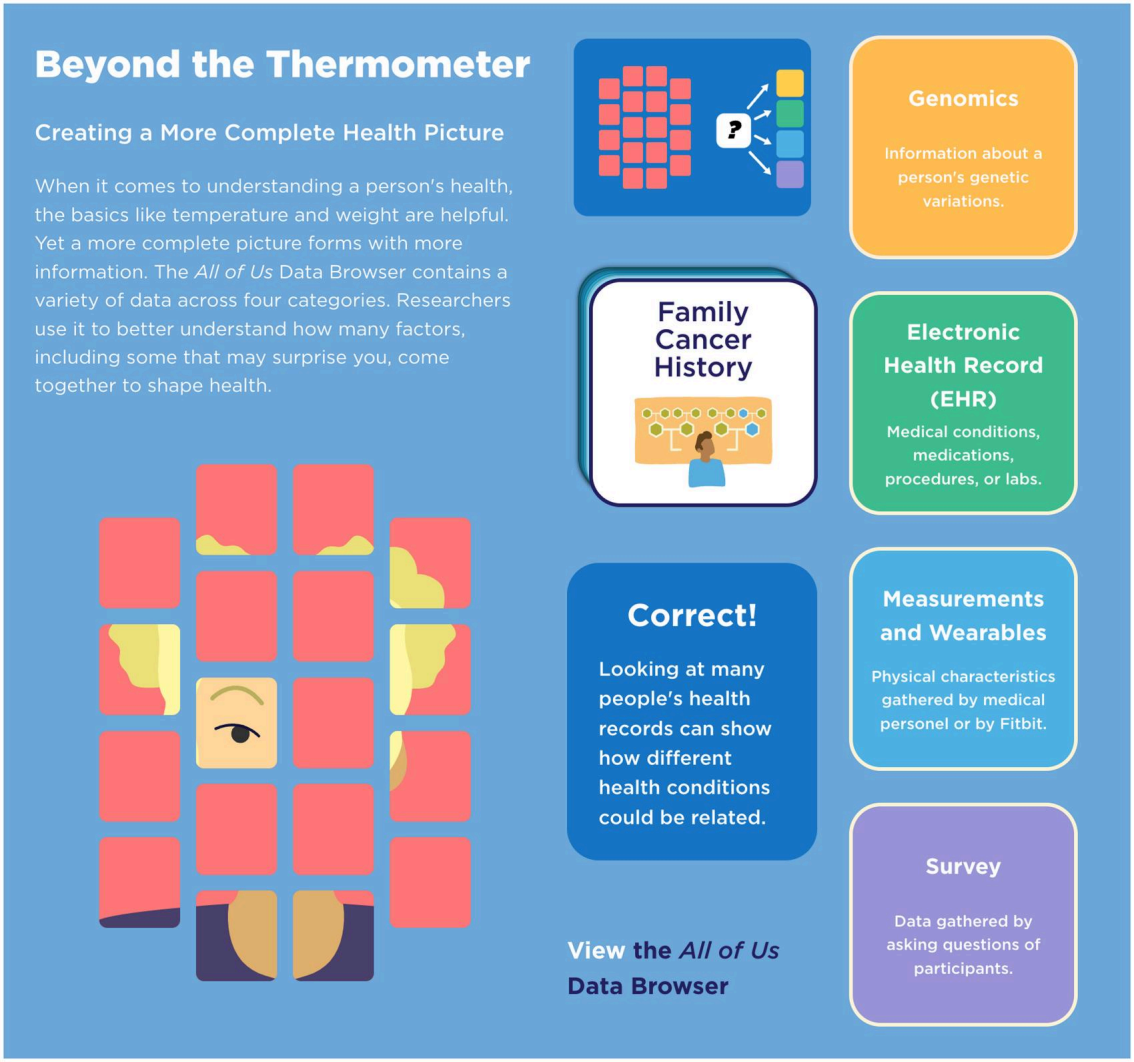
In the “Beyond the Thermometer” web-based interactive activity, students sort health-related data types into the categories in which they are found in the Data Browser. As more types of data are correctly identified, a more complete picture of a person emerges. The game randomly generates sketches of 14 people who represent the diversity of participants in the *All of Us* Research Program.

**Figure 5.**
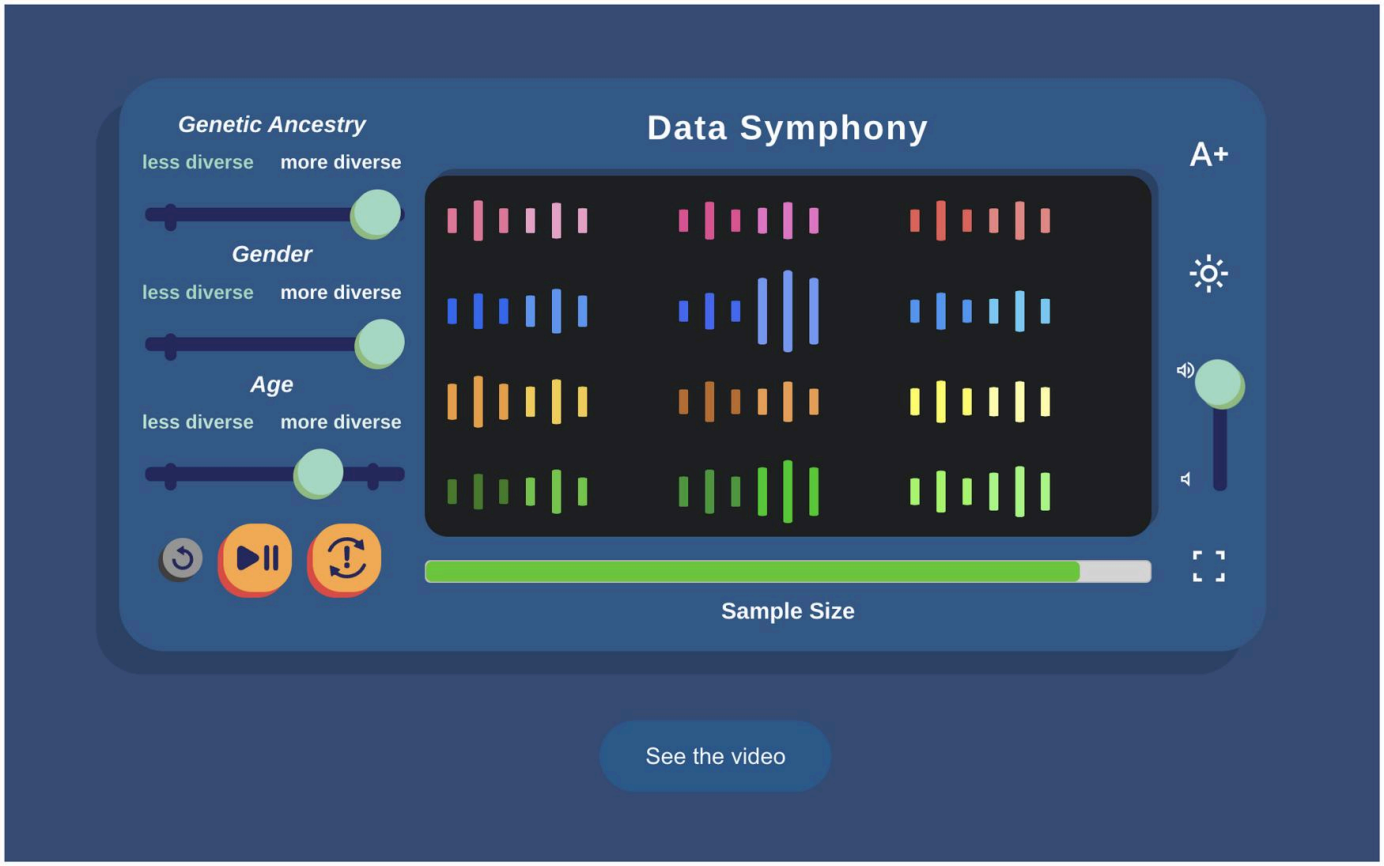
Students are the composer for the “Data Symphony”! Through an interactive musical interface, they manipulate the amount of diversity to see and hear how much richer the music is when more data is added. They also learn that data inaccuracies fall away with large datasets.

**Figure 6.**
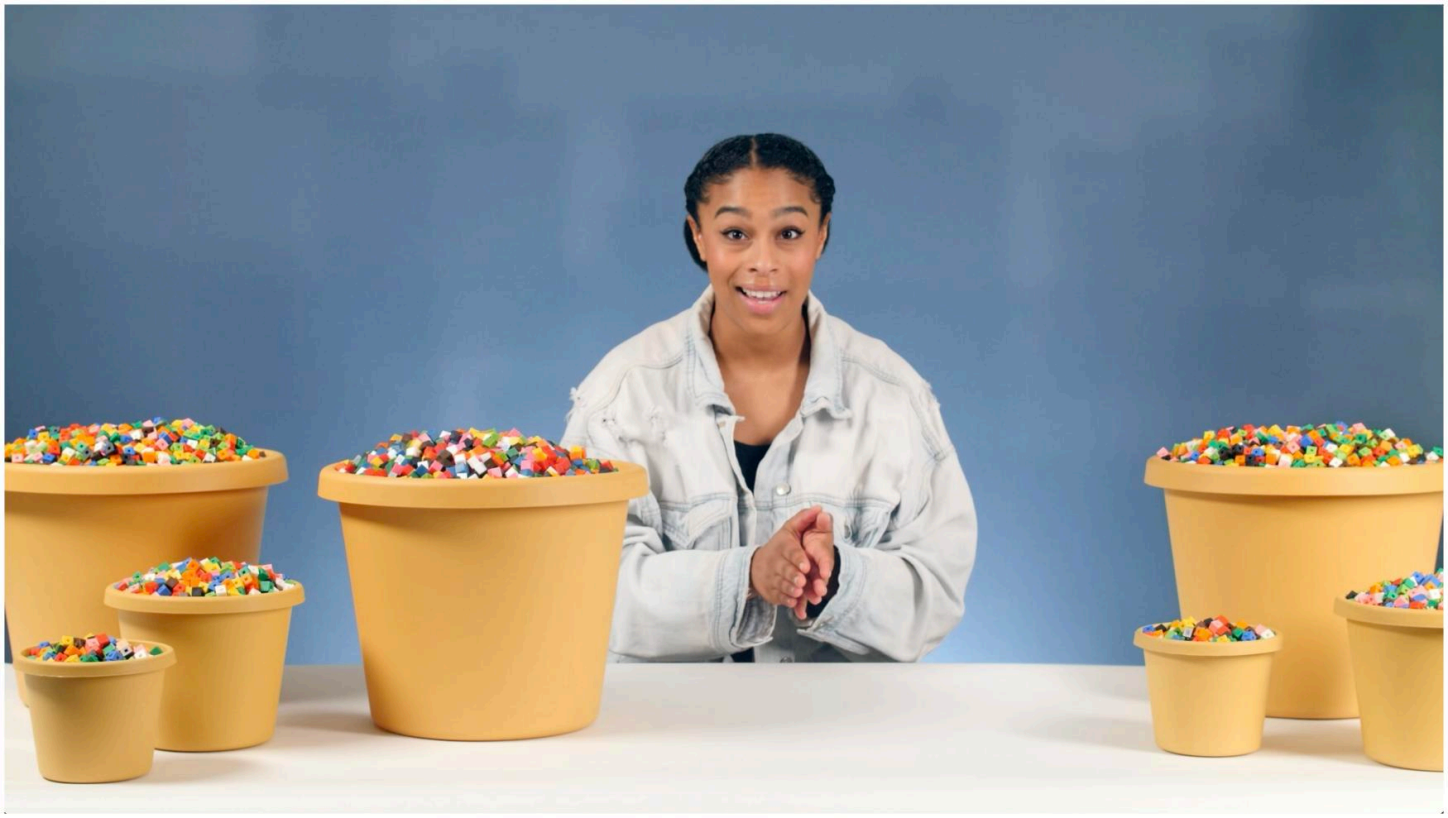
In the “What is Big Data?” video, students learn that (a) “big data” is a term used to describe large amounts of data that can be mined for information, and (b) computers and specialized programs are needed to store, process, and analyze data on a large scale. Video screen shot used with permission from the Genetic Science Learning Center.

The multimedia activities remain rooted in the Data Browser but also expand into broader applications. In “Beyond the Thermometer” (Figure 4), students explore how the types of data collected by *All of Us* create a more complete picture of human health; a paper-based version also is available to download and print. The “Data Symphony” (Figure 5) is an interactive piece that combines music and science. Students adjust the amount and diversity for 3 types of data found in the Data Browser to see and hear how different the resulting music can sound. A built-in walkthrough video makes the piece usable for those who prefer to experience it as a primarily audio experience. Finally, the “What is Big Data” video (Figure 6) pulls together several positive examples, from biomedical research and beyond.

The teacher support materials for the module provide a brief description of each “activity” as well as learning objectives, estimated class time, downloadable PDFs for print-based activities, and class discussion guides. Suggested learning sequences provide ideas for using the module activities to engage students in exploring each of 3 questions: (1) What is the *All of Us* Research Program?, (2) Why does sample size matter?, and (3) How is big data useful for research?

### Classroom implementation pilot tests

Analysis of data collected during the Phase 1 pilot of the Data Browser curriculum and assessment involved both quantitative and qualitative methodologies. Statistical analyses assessed answer variability, item reliability, and difficulty estimates. Transcripts from the cognitive think-aloud interviews were analyzed to identify problematic items and areas of confusion as reported by students. These data, along with recommendations from *All of Us* staff, informed revisions, and the ultimate selection of 22 items from the original 30 for the assessment instrument.

Following completion of the Phase 2 pilot, statistical analyses were used to examine answer variability, reliability, and difficulty estimates to determine the degree to which the 22 selected items continued to function as anticipated. Based on these analyses, no items were identified for removal. This finalized instrument will be used for Phase 3 of the study, the national field test, which is being conducted in spring 2024. This phase will evaluate the degree to which a large sample of students who participate in the *All of Us* Data Browser curriculum demonstrate mastery of the intended learning outcomes.

Data from the Phase 1 classroom pilot student assessments indicated that the 34 students who took form A of the assessment and who were retained in the dataset correctly answered 9.75 out of 15 questions, 65%, on average. On average the 19 students who completed form B correctly answered 66%, or 9.9 out of 15 questions. Responses for 214 students were analyzed from the Phase 2 assessment. On average, these students answered 21 out of 22 questions, with an average score of 71% correct for the questions they answered.

The assessment items performed similarly in both Phase 1 and Phase 2. Students demonstrated a strong understanding of the main types of health data available in the *All of Us* Data Browser, with 86% identifying them correctly. However, they faced the greatest difficulty in recalling that the Data Browser also includes data on environmental pollutant exposure, with only 55% answering this question accurately. Students demonstrated understanding of the definition of “big data” (82% correct) and the necessity of computers for working with big data (86% correct). Additionally, over 80% of students correctly answered questions about the importance of data diversity and sample size, suggesting that the curriculum materials were largely effective in attaining the aims defined in the learning objectives at the expected Depth of Knowledge.

### Teacher feedback on the curriculum materials

The survey soliciting feedback on the Data Browser materials was completed by 7 of the 19 teachers who participated in the Co-design Summer Institute. Overall, 71%-100% of these teachers rated each activity as “Extremely useful” or “Very useful” (Table 3). Example feedback from open-response questions is shown in Table 4. Teachers noted their satisfaction with the curriculum overall and their contributions, with one teacher writing “I was happy to see how the work we did in the summer institute translated into a working piece of curriculum! So cool to see my group’s input.”

**Table 3.** Co-design Summer Institute teachers’ responses to survey questions on the usefulness of incorporating each activity in the Data Browser module into their instruction.

Activity	Extremely useful	Very useful	Moderately useful	Slightly useful
Data Browser Webquest	42.9% (3)	42.9% (3)	0%	14.3% (1)
How Big is Your Data?	42.9% (3)	28.6% (2)	28.6% (2)	0%
Research with the Data Browser	14.3% (1)	85.7% (6)	0%	0%
Beyond the Thermometer interactive	28.6% (2)	42.9% (3)	28.6% (2)	0 %
Data Symphony interactive	28.6% (2)	42.9% (3)	14.3% (1)	14.3% (1)
What is Big Data? video	57.1% (4)	42.9% (3)	0%	0%

**Table 4.** Representative examples of feedback about the *All of Us* Data Browser curriculum materials from surveys completed by Co-design Summer Institute and pilot test teachers.

***Data Browser Webquest* print-based data exploration guide**
“The web quest will allow me to introduce data to my students in a meaningful way. Students can explore various points of data in a structured way that supports individual exploration.” (Summer Institute teacher)
***How Big is Your Data?* print-based data exploration guide**
“This was really fun because they were up doing, which they love. I like the idea of doing this with blood pressure as well, and we may do that next year with our kiddos, too! I had my students create 2 graphs: one of data from their tiny groups only, and one from the whole class. Then to compare those to the *All of Us* browser data was a really great way to wrap up the whole unit!” (Pilot test teacher)
***Research with the Data Browser* print-based data exploration guide**
“[This activity] can be used in so many ways! The students are able to formulate their own research question, which is a vital part of the science curriculum. They are also able to look at raw data and draw conclusions.” (Summer Institute teacher)
***Beyond the Thermometer* interactive**
“As I walked around the room, students were highly engaged with this activity and often referred to the *All of Us* website to check their work (they were invested and wanted to get the correct answer). This seemed to be an effective way to guide students in exploring a certain section of the *All of Us* Data Browser.” (Pilot test teacher)
“The activity can maybe have more info on the card being sorted. Some students may not know what it is referring to.” (Summer Institute teacher)
***Data Symphony* interactive**
“I loved Data Symphony. What a beautiful interactive! I most liked having the students think about the power of this project and how it can change things for us.” (Pilot test teacher)
“The Data Symphony is an excellent way to explain [to] students why big and diverse data is important by comparing it with a symphony. Having big and diverse data analytics provides the ability to identify patterns, trends, and anomalies that may not be apparent with a small and non-diverse data [set].” (Summer Institute teacher)
“I don’t think this analogy worked very well. …I was not familiar with this symphony to be able to tell when the tune is missing something. Maybe do a familiar tune so it starts to sound more familiar as the data become more diverse.” (Summer Institute teacher)
***What is Big Data?* video**
“The video was engaging for all students and the short length was able to hold their attention. We followed up as a whole class discussing other potential uses of big data and they were able to come up with good examples, such as how ACT test data or online algorithms can be used.” (Pilot test teacher)
“I think the video would be a great introduction (or review) for learning about data collection.” (Summer Institute teacher)
**Overall**
“It's a great way to get your students interested in the possibilities of the *All of Us* project, and to think about the kinds of questions they might pursue.” (Pilot test teacher)
“I think that these activities will allow many levels of learners to interact with the big data. The videos are short and cohesive which will keep the students’ interest.” (Summer Institute teacher)
“A detailed sequence of lesson plans along with alignment of these activities with health and science standards would be helpful to teachers.” (Summer Institute teacher)
“I would make these activities more accessible to English Language Learners. I would give students the option to select their language in the student sheet fillable PDF and would make the video with a closed caption option.” (Summer Institute teacher)

Data from the Phase 1 pilot test teachers’ post-instruction surveys informed improvements to the instructional guidance for teachers prior to the Phase 2 pilot test. Survey data from the Co-design Summer Institute teachers and the post-instruction survey data from the Phase 2 pilot test teachers provided additional recommendations. Those that could be addressed within time and funding constraints were incorporated prior to the beginning of the national field test. Table 4 presents selected quotes from all surveys that highlight key aspects of teachers' feedback and experiences regarding the curriculum materials.

## Discussion

The teachers who participated in the Co-design Summer Institute brought their experience with multiple courses, grade levels, and range of student preparation for engaging in research. This enabled us to collaboratively develop a set of curriculum materials that are flexible and can be used in multiple settings. On the end-of-Institute survey, participating teachers listed 15 different courses in which they might use the Data Browser materials including several types of biology, biotechnology, anatomy and physiology, biomedical science, medical terminology, health, social studies, chemistry, environmental science, general science, forensics, computer programming (coding), math, research, and ACT prep.

There was some difference of opinion between participants’ strong agreement in the value of developing curriculum materials utilizing the Data Browser and their slightly less strong support for its worth as a teaching tool. This may be due to limitations in the data that are currently publicly available. For example, one participant noted “The first ideas I have for using the data within a classroom were limited by what is available to the general public on the Data Browser. But the topic of big data in general and inclusivity are worth introducing the students to.” *All of Us* continues to evaluate which data features to make available on the Data Browser without compromising the privacy and security of participants’ data. These improvements mean that the paper-based curriculum materials may need to be updated as the program grows and evolves.

In their survey responses, teachers who participated in the Co-design Summer Institute and the pilot tests provided positive feedback on the Data Browser curriculum materials as well as suggestions for expansion or improvement. For example, several teachers recommended adaptations that would make the activities more accessible to English Language Learners. These provide guidance for ways in which the materials could be expanded upon when additional funding becomes available.

### Study limitations

Only 7 (37%) of the teachers who participated in the Co-design Summer Institute responded to the survey soliciting their feedback on the Data Browser module activities. Input from more participants would have provided additional information on the perceived usefulness of the materials and for improving them.

Although a 2-phase validation strategy was implemented (pilot testing with a limited sample followed by a larger national sample), further validation with a more extensive and diverse population would be beneficial. This would ensure that the assessment items perform effectively across a broad spectrum of student backgrounds and abilities. To strengthen the validation process, conducting more comprehensive analyses of item difficulty, discrimination, and reliability, as well as exploring the assessment's predictive validity concerning other measures of student achievement, would be advantageous. Nevertheless, the current validation process establishes a solid foundation for utilizing the assessment to evaluate student learning outcomes associated with the *All of Us* Data Browser curriculum materials.

We did not solicit written feedback from students who used the Data Browser materials, instead relying on teachers’ reports of students’ responses to the materials. This reduced the class time needed for pilot testing but did not provide an opportunity to hear directly from students.

## Conclusion

The *All of Us* Research Program provides a unique opportunity for secondary students to engage in exploring a large, diverse dataset to learn about the value and applications of “big data” in biomedical research. By collaborating with secondary teachers to co-design curriculum materials that utilize the *All of Us* Data Browser, we were able to develop engaging, accessible learning experiences that fit the needs of secondary classrooms. Pilot testing across participating schools showed substantial growth in students' understanding of key big data concepts and research applications. Our co-design process and curriculum serve as a model for introducing students to precision medicine research by exploring diverse real-world datasets. Providing early educational experiences with large datasets like *All of Us* can help cultivate data literacy skills and interest in biomedical research careers among the next generation of potential researchers and engaged citizens. Further research should examine the effectiveness of this approach in post-secondary educational settings and its potential to provide a bridge to more advanced use of the *All of Us* Researcher Workbench.

## Data Availability

Interested parties may contact Rebecca J. Peterson, PhD, at the University of Utah with data requests: rebecca.peterson@utah.edu
